# A population-based study of the association between food insecurity and potentially avoidable hospitalization among persons with diabetes using linked survey and administrative data

**DOI:** 10.23889/ijpds.v4i1.1102

**Published:** 2019-08-05

**Authors:** N Gupta, Z Sheng

**Affiliations:** 1 Department of Sociology, University of New Brunswick, PO Box 4400, Fredericton, New Brunswick E3B 5A3, Canada

**Keywords:** Data linkage, health surveys, hospital records, diabetes mellitus, hospitalization, food security

## Abstract

**Background:**

Studies have found food insecurity to be more prevalent among persons with diabetes mellitus. Other research using areal-based measures of socioeconomic status have pointed to a social gradient in diabetes hospitalizations, but without accounting for individuals’ health status. Linking person-level data from health surveys to population-based hospital records enables profiling of the role of food insecurity with hospital morbidity, focusing on the high-risk diabetic population.

**Objective:**

This national study aims to assess the association between income-related household food insecurity and potentially avoidable hospital admissions among community-dwelling persons living with diagnosed diabetes.

**Methods:**

We use three cycles of the Canadian Community Health Survey (2007, 2008, and 2011) linked to multiple years of hospital records from the Discharge Abstract Database (2005/06 to 2012/13), covering 12 of Canada’s 13 provinces and territories. We apply multiple logistic regression for testing the association of household food insecurity with the risk of hospitalization for diabetes and common comorbid ambulatory care sensitive conditions among persons aged 12 and over living with diabetes.

**Analysis:**

Data linkage allowed us to analyze inpatient hospital records among 10,260 survey respondents with diabetes; 590 respondents had been hospitalized at least once for diabetes or a common comorbid chronic physical or mental illness. The regression results indicated that the odds of experiencing a preventable hospital admission were significantly higher among persons with diabetes who were food insecure compared to their counterparts who were food secure (OR=1.66 [95%CI=1.24-2.23]), after controlling for age, sex and other characteristics.

**Conclusion:**

We found food insecurity to significantly increase the odds of hospital admission for ambulatory care sensitive conditions among Canadians living with diabetes. These results reinforce the need to consider food insecurity in public health and clinical strategies to reduce the hospital burden of diabetes and other nutrition-related chronic diseases, from primary prevention to post-discharge care.

## Introduction

The prevalence of diabetes mellitus continues to increase in Canada and around the world. Globally, the number of adults with diabetes quadrupled between 1980 and 2014, a trend attributed in part to population growth and population aging [1]. In Canada, different data sources have been used to assess trends in the numbers of persons with diabetes. These include data from administrative health records and from population-based surveys, some of which rely on self-reports. Figures from population-based administrative data collated through the Canadian Chronic Disease Surveillance System indicate the national age-standardized diabetes prevalence rate increased by 70% between 1999 and 2009 [2]. Diabetes prevalence has been found to be higher among men than women, and among those in lower income groups compared to more affluent Canadians, as assessed using self-reports from the Canadian Community Health Survey (CCHS) [3]. Based on blood samples collected through the Canadian Health Measures Survey, Rosella et al. estimated that at least 20% of type 2 diabetes cases remain undiagnosed [4].

A complex disease, diabetes is associated with wide-ranging adverse physical health consequences, including a number of cardiac, vascular, pulmonary, genitourinary, and reproductive conditions, as well as mental health disorders. Many of the common physical comorbidities of diabetes share underlying predisposing factors and a common management plan, known as diabetes-concordant conditions [5,6,7]. In Canada, people with diabetes are hospitalized on average three times as often for cardiovascular disease and twelve times as often for kidney failure, and associated public healthcare costs are three to four times higher, compared to those without diabetes [2]. While the causal direction of mental illness and diabetes is not clear, there is growing recognition that depression and other mood and anxiety disorders are a complication of diabetes [2,8]. Hospitalizations for diabetes and many of its chronic physical and mental comorbidities are considered ambulatory care sensitive, that is, potentially avoidable through appropriate management in primary care and by acting on the key underlying patient factors, such as socioeconomic status, that may place individuals at greater risk of hospitalization [9,10,11].

The existence of a graded association between socioeconomic status and health across population subgroups has been widely documented [12,13]. Recent systematic reviews have highlighted evidence that low socioeconomic status is related to higher morbidity and poorer quality of clinical care for patients with both types 1 and 2 diabetes, and this even in systems of universal healthcare coverage [14,15]. Evaluations of socioeconomic inequality and diabetes outcomes employ a variety of study designs, and are generally distinguished between those considering individual- versus regional-level deprivation measures, mostly based on characteristics of the data source used [14].

Identified pathways from socioeconomic inequality to health include availability of food and other societal investments in human capital development [13]. A previous study using CCHS data for selected Canadian jurisdictions found household food insecurity to be more prevalent among persons with diabetes compared to those without the disease [16]. Food insecurity was associated with low consumption of vegetables and fruits, although higher consumption is clinically recommended as beneficial for glycaemic control and reducing the risks of development and progression of diabetes complications. High prevalence of food insecurity among patients with diabetes has also been found in resource-limited settings, despite the well-known contribution of high caloric intake and obesity to the development of type 2 diabetes [17]. However, population-based studies with large samples to clarify how food insecurity affects long-term complications and health outcomes among individuals with diabetes remain limited [18].

The objective of this study is to investigate the relationship of income-related food insecurity with the risk of severe morbidity and preventable hospitalization attributed to diabetes and associated chronic comorbidities among persons with diagnosed diabetes. We use a large-scale data resource at the population level: household health surveys linked to administrative records on hospital inpatient care. We consider all hospital admissions for diabetes (types 1 and 2) and its main comorbid ambulatory care sensitive conditions (ACSCs). Previous assessments of the risk of hospitalization for chronic ACSCs have often been hampered by a failure to account for health status [19]. The use of linked survey and administrative data sets help us to overcome this shortcoming, thereby presenting a novel research path for assessment of the social gradient in diabetes and health.

## Methods

Our study draws on linked population-based survey and administrative data, and conforms to the *REporting of studies Conducted using Observational Routinely-collected health Data* (RECORD) protocol [20].

### Data sources

We use multiple years of person-level health survey data linked to multiple years of administrative inpatient hospital discharge records to attain sufficient sample sizes of hospital admissions for our target population: persons ages 12 and over who report having been diagnosed with diabetes. The combined estimates reflect an average risk of potentially avoidable hospitalization roughly corresponding to the period 2006-2011. Details on the microdata linkage process are documented elsewhere [21,22]. In brief, a probabilistic approach was used on given and family names, birthdate, sex, and postal code of residence among survey respondents who agreed to link their information to other databases. Approximately 85% of respondents agreed to link their data; results vary across survey years [23]. The record linkage enables the creation of patient histories by counting hospitalizations (including diagnostics and interventions) at the person level.

#### Canadian Community Health Survey (CCHS)

The CCHS is cross-sectional survey program administered by Statistics Canada. It collects information on health determinants, health status, and health care by means of personal and telephone interviews from a nationally representative sample of the community-dwelling population aged 12 and over at the time of the survey. The sampling frame does not include people residing on Aboriginal reserves, on Canadian Forces bases, in institutions, or in some remote areas (representing ~2% of the total population).

The CCHS contains a core set of questions asked to all respondents every year, as well as thematic content which alternates from year to year, or is only asked in some provinces and territories depending on local information priorities. In particular, the core chronic disease module comprises a series of questions using validated algorithms designed to identify respondents with physician-diagnosed diabetes mellitus [24]. The data are widely used to monitor the prevalence of diabetes in Canada, but are not clinically validated to distinguish the different types of diabetes [25].

To address our research objective, we also take advantage of the thematic Household Food Security Survey Module (HFSSM), which focuses on self-reported inadequate food access due to limited financial resources [26]. The module does not assess other dimensions of food security, such as the availability of culturally preferred foods [16]. Since the HFSSM is not included every year in every province and territory, we use only three CCHS cycles within the period of observation with nationally representative data on food insecurity as core survey content: 2007, 2008, and 2011. Food insecurity rates remained stable in Canada between 2007 and 2011 [27], so it is unlikely our estimates reflect significant changes over time at the population level for this period.

#### Discharge Abstract Database (DAD)

The DAD contains administrative, clinical, and demographic information on all acute-care and some psychiatric, chronic, rehabilitation, and day-surgery hospital discharges within a fiscal year (the period from April 1 of a given year to March 31 of the following year), covering 12 of Canada’s 13 provinces and territories (excluding Quebec) [28]. Thanks to a single-payer universal healthcare system, the data are considered essentially complete for reporting jurisdictions.

Since 2005, diagnostic data in DAD records have been consistently coded to the International Classification of Diseases, 10th revision, Canadian adaptation (ICD-10-CA) [29]. To ensure adequate sample sizes of hospitalizations for diabetes and comorbid ACSCs, we include all discharge databases from 2005/06 through 2012/13 in our analysis. Since individuals may experience more than one hospitalization, we searched all records for any stay with a most responsible diagnosis of diabetes or selected physical and mental health ACSCs. Diagnoses of types 1 and 2 diabetes (including complications related to hypo- or hyperglycaemia) as the main reason for a hospital stay during the period of observation were identified through the ICD-10-CA codes E10.x-E14.x. A study by Jiang et al. indicated high validity of diabetes coding algorithms in the DAD compared to a prospective clinical registry [30].

We also flagged any hospitalization where the most responsible diagnosis was for any of the following chronic physical and mental health ACSCs commonly comorbid with diabetes: obesity (ICD-10-CA codes E66.x), hyperlipidemia (E78.x), hypertension (I10.x-I15.x), ischemic heart disease (I20.x-I25.x), cardiomyopathy (I42.x-I43.x), cardiac arrhythmia (I47.x-I49.x), congestive heart failure (I50.x), stroke (I60.x-I64.x), coronary atherosclerosis (I70.x), thromboembolism (I80.x-I82.x), chronic obstructive pulmonary disease (J41.x-J45.x), chronic kidney disease (N18.x), and polycystic ovarian syndrome (E28.2) as well as mood and anxiety disorders (F30.x-F48.x). While different studies and healthcare organizations use different categorizations of what is considered an ACSC, these conditions have been considered to reflect common coexisting physical and mental health disorders among persons with diabetes relevant for investigations of multimorbidity, care interventions, and preventable hospitalizations [7,8,31].

### Statistical analysis

Our target population is persons aged 12 and over living with diabetes, as identified in the CCHS share link microdata file. Our outcome of interest is ever-admitted for diabetes or a comorbid ACSC during the period of observation. Following a presentation of descriptive statistics, we use multiple logistic regression analysis to examine the associations between household food insecurity and potentially avoidable hospitalization. We further control for age group, sex, body mass index, and province or region of residence as potential confounding factors. Characteristics at the time of the survey are assumed to be those at the time of the hospitalization.

To facilitate interpretation of the results from the logistic regression, we generate adjusted odds ratios (ORs) and the associated 95% confidence interval (95% CI) for each risk factor. We limited the samples to survey respondents with non-missing data for the variables of interest. All counts were rounded to a base of five, and adjusted to reinforce the confidential nature of the data using Statistics Canada control algorithms. Survey weights were applied to the descriptive statistics to ensure population representation of the findings. Analyses were conducted using the Stata 15 statistical software package [32].

### Accessibility of data and materials

We accessed the de-identified linkable data sets in the secure environment of the New Brunswick Research Data Centre (RDC), which is part of the Canadian Research Data Centre Network. Researchers wishing to access these and other confidential data and documentation from Statistics Canada may submit a project proposal through the RDC program [33]. Non-confidential CCHS questionnaires and public use microdata files are available through the Data Liberation Initiative [34]. This study complied with the University of New Brunswick’s Research Ethics Board, which does not require an internal institutional review for research projects using data accessed through the RDC.

## Results

### Study population selection

Based on the CCHS data, our target subgroup included 10,260 persons living with diabetes, or 7.7% of a total of 133,245 respondents aged 12 and over for the three combined survey cycles (2007, 2008, and 2011), excluding residents of Quebec. Fewer than 0.1% of all respondents did not report their diabetes status.

**Figure 1: Flow chart for the creation of analysis file from linked health survey and hospital data:**
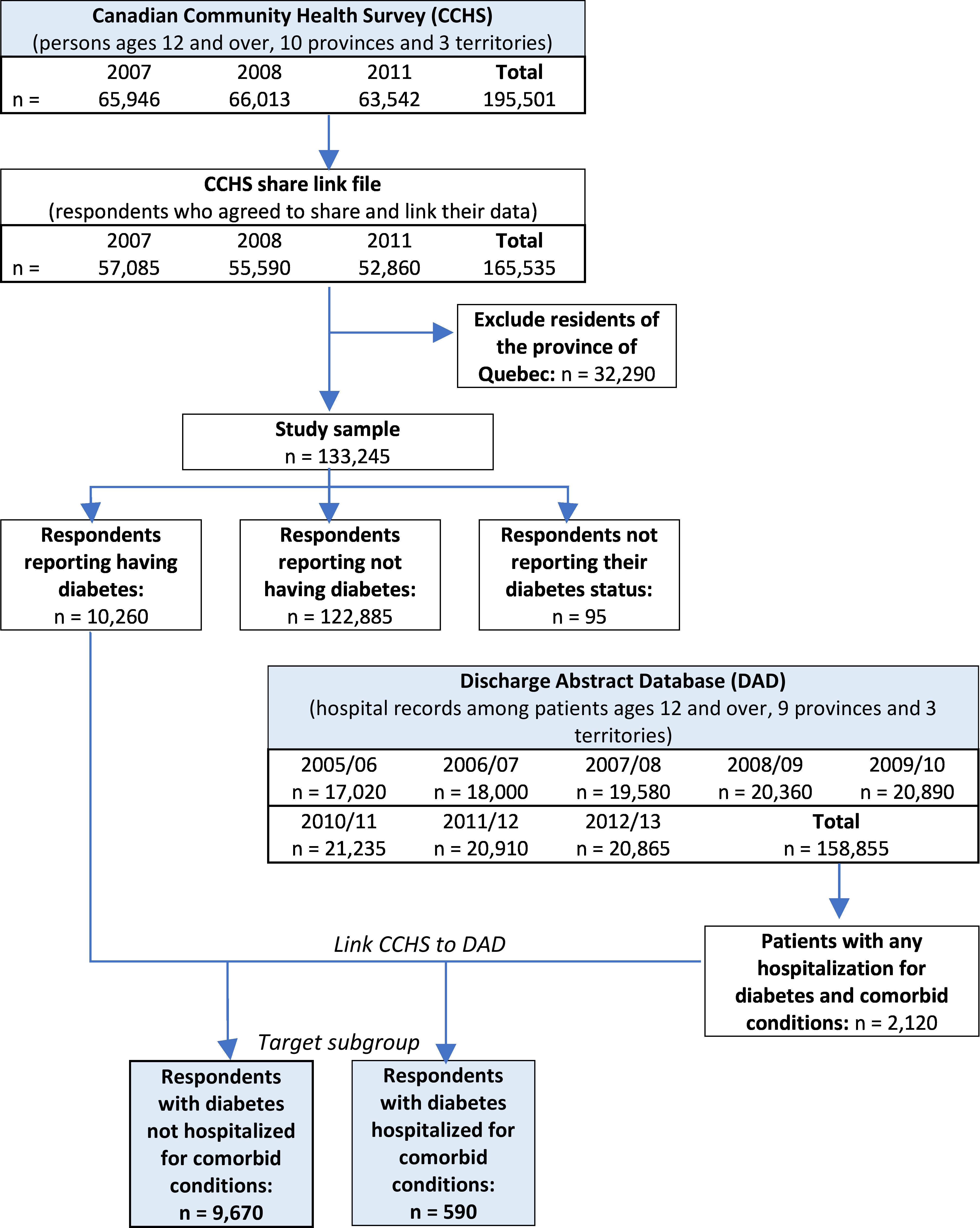
Note: Numbers may not add due to control rounding.

According to the DAD data, 2,120 persons eligible to be linked to the CCHS had been hospitalized at least once with primary diagnosis of diabetes or a comorbid ambulatory condition in the period of observation (out of 158,855 discharge records analyzed). The bulk of these hospitalizations were for comorbid conditions. Following data linkages, among the target survey sample living with diabetes, 590 respondents had experienced at least one hospitalization for diabetes or a comorbid condition. A flow chart of the study population selection is found in Figure 1.

### Study population description

As seen in Table 1, 10.5% of the community-dwelling population aged 12 and over living with diabetes experienced income-related food insecurity. As expected, persons with diabetes tended to be overrepresented in the older age groups and among males. Additionally, 4.8% of persons with diabetes had experienced a hospitalization for diabetes or a comorbid physical or mental health condition in the period of observation.

**Table 1: Percentage distribution of the community-dwelling population aged 12 and over living with diabetes by selected characteristics, Canada, 2006-2011:** Note: Data weighted to ensure population representation. Source: Linked CCHS and DAD data (excluding Quebec).

Household food security status		
	Food secure	89.5%
	Moderately/severely insecure	10.5%
Age group		
	12-24 years	1.9%
	25-44 years	11.3%
	45-64 years	44.9%
	65+ years	41.9%
Sex		
	Female	45.0%
	Male	55.0%
Province/region of residence		
	Atlantic provinces	12.6%
	Ontario	53.1%
	Prairie provinces	18.7%
	British Columbia and territories	15.6%
Hospitalized at least once for diabetes or a comorbid condition		
	Yes	4.8%
	No	95.2%

Sample size (unweighted)		10,260

Bivariate analysis revealed persons with diabetes who were hospitalized at least once for this disease or a comorbid condition in the period of observation had a significantly higher rate of food insecurity compared to those who were not hospitalized (χ^2^=4.594; p=0.032).

### Multivariate analysis of the association between food insecurity and preventable hospitalization

Results from the multiple logistic regression indicate that the odds a person with diabetes being admitted to hospital at least once for this disease or its common comorbid conditions were significantly higher among those who experienced income-related food insecurity compared to their counterparts who were food secure (OR=1.66 [95%CI=1.24-2.23]), after controlling for age group, body fatness and other characteristics (Table 2).

Compared to adults aged 25-44 years, those aged 45-64 years were nearly twice as likely to be hospitalized (OR=1.75 [95%CI=1.08-2.84]), and those aged 65 and over approximately three times as likely to be hospitalized (OR=3.04 [95%CI=1.89-4.88]), all else being equal. Adolescents and young adults were also more likely to be admitted to hospital, likely for acute morbidity attributable to type 1 diabetes. Being female and having a lower body mass index were each slightly protective of the risk of hospitalization, but the associations were not statistically significant.

**Table 2: Adjusted odds ratios (and 95% confidence intervals) for the risk of hospitalization for diabetes or a comorbid condition among community-dwelling persons living with diabetes:** Note: * = p<0.05; ref = reference category. Source: Linked CCHS and DAD data (n=10,260).

		Odds ratio	Confidence interval

Household food security status			
	Food secure (ref)	1.00	..
	Moderately/severely insecure	1.66*	1.24 - 2.23
Age group			
	12-24 years	5.08*	2.45 -10.53
	25-44 years (ref)	1.00	..
	45-64 years	1.75*	1.08 - 2.84
	65+ years	3.04*	1.89 - 4.88
Sex			
	Female (ref)	1.00	..
	Male	1.09	0.92 - 1.31
Body mass index			
	BMI (kg/m2)	0.99	0.98 - 1.01
Province/region of residence			
	Atlantic provinces	1.06	0.83 - 1.35
	Ontario (ref)	1.00	..
	Prairie provinces	1.38*	1.10 - 1.72
	British Columbia and territories	1.17	0.89 - 1.53

## Discussion

A growing body of literature is providing insights on the association between food insecurity and diabetes outcomes, including worse health status, medication adherence, self-care practices, and behavioural risk factors [16,18,35]. Persons with diabetes who experience food insecurity may have limited control over what they eat, which may lead to poor glycaemic control and, in turn, severe morbid events [35]. However, there is a limited research on this pressing public health issue using large-scale data sets at the population level. Innovative strategies to improve the availability and use of linkable nationally-representative datasets with comprehensive measures of food insecurity, disease status, and healthcare utilization are helping to build the evidence base on the relationships between them [36].

We found that one in ten (10.5%) of Canadians aged 12 and over living with type 1 or type 2 diabetes experienced income-related food insecurity. This proportion was higher than the rate of food insecurity among the general population of the same age range, which was 8.4% in 2011 [27]. Our national assessment using linked population-based survey and clinical data found significantly higher odds of potentially avoidable hospitalization for diabetes or a comorbid ambulatory care sensitive physical or mental health condition among Canadians with diabetes who were experiencing income-related food insecurity compared to their counterparts who were food secure, after controlling for age, body mass index and other characteristics. These results reinforce the need to integrate food security considerations across the continuum of care for persons with diabetes, from primary to post-discharge care. A pilot study from Canada suggested that routine food insecurity screening is acceptable to both patients and clinicians, and can help increase understanding of why some patients may not follow diabetes self-management recommendations [37].

Certain limitations to our study should be noted. First, while we characterized our study as national in scope, coverage of the linkage between the CCHS and hospital records is incomplete [38]. Notably, the DAD data exclude records from Quebec. While levels of household food insecurity among the Quebec population were not statistically different from the national average in 2011 [27], given the exclusion from the CCHS sampling frame of Aboriginal people living on reserves and people living in some remote regions, we may be underestimating the true prevalence of food insecurity across the country. Survey data have previously revealed the food insecurity rate to be more than three times as high among off-reserve Aboriginal households compared with non-Aboriginal households [26]. Food insecurity is widely recognized as a particularly serious challenge in Canada’s northern and remote Aboriginal communities, but there remains a dearth of evidence on the health impacts among this vulnerable group, which is simultaneously more susceptible to diabetes and other nutrition-related chronic conditions [39].

Second, the DAD data exclude emergency department visits that did not result in admission. A retrospective study from the United States found that, after controlling for demographics and health insurance, food insecurity was associated with significantly more all-cause emergency department visits among a national cohort [40]. More research is needed as to whether food insecurity is related to overall hospital service utilization and associated healthcare costs among persons with chronic disease.

Third, as inherent to studies of a cross-sectional nature, we cannot draw causal inferences from our results. We estimated the average risk of potentially avoidable hospitalization roughly corresponding to the period 2006-2011, linking routinely collected administrative hospital data with periodically collected survey data. It is possible that, for example, some individuals may have experienced loss of earned income and food insecurity as a result of being hospitalized. Some patients captured in a DAD record prior to CCHS interview may have died or been transferred to a long-term care facility, thus becoming ineligible for data linkage. Some may have received their diabetes diagnosis during a hospital stay subsequent to interview. Underestimates of diabetes prevalence from self-reported survey data have been observed elsewhere [41]. Studies have also suggested that adults living in food-insecure households may be less likely to report a diagnosis of diabetes [42].

Diabetes-attributable hospitalizations are relatively rare, statistically speaking. Patients with diabetes are hospitalized more often for comorbid complications than directly for diabetes. While we considered a number of comorbid physical and mental health conditions, and used national-level data, the numbers of hospital stays remained small. That said, this study using linked survey and clinical data represents, to the best of our knowledge, the first Canadian population-based assessment of the role of food security as a social determinant of potentially avoidable hospitalization among Canadians with diabetes across the life span.

## Conclusion

The evidence base is growing on the association between socioeconomic status and differential risk of hospitalization across population groups, including in contexts of universal healthcare coverage such as Canada. Previous studies have tended to consider socioeconomic status as a predictor of preventable hospitalization in terms of personal income, neighbourhood income level, educational attainment, or employment status. Taking advantage of linked national survey and administrative data, our study uniquely looked at the role of food insecurity as a barrier to improved health outcomes. Some studies have shown food insecurity to be related to higher rates of numerous chronic diseases including diabetes. We found income-related food insecurity to significantly increase the odds of hospitalization for diabetes and its common comorbid complications among Canadians living with diagnosed diabetes compared to those who were food secure (OR=1.66 [95%CI=1.24-2.23]). These results reinforce the need to consider food insecurity in public health and clinical strategies to reduce the hospital burden of diabetes.

## List of abbreviations

ACSC	ambulatory care sensitive condition
CCHS	Canadian Community Health Survey
CI	confidence interval
DAD	Discharge Abstract Database
HFSSM	Household Food Security Survey Module
ICD-10-CA	International Classification of Diseases, 10th revision, Canadian adaptation
OR	odds ratio
RDC	Research Data Centre
RECORD	REporting of studies Conducted using Observational Routinely-collected health Data
